# Supplementary material for: Integrative Metabolomics and Proteomics Detected Hepatotoxicity in Mice Associated with Alkaloids from Eupatorium fortunei Turcz
Source: Toxins (Basel). 2022 Nov 5;14(11):765. doi: 10.3390/toxins14110765 (PMC9698670; doi:10.3390/toxins14110765)
Supplement: Supplementary file 1 [file toxins-14-00765-s001.zip › toxins-1968335-supplementary.pdf]

toxins

MDPI

SUPPLEMENTARY MATERIALS

Integrative Metabolomics and Proteomics Detected Hepatotoxicity in Mice Associated with Alkaloids from *Eupatorium fortunei* Turcz.

Ke Zan [1], Wei Lei [2,*], Yaolei Li [1], Ying Wang [1], Lina Liu [1], Tiantian Zuo [1], Hongyu Jin [1] and Shuangcheng Ma [1,*]

[1] National Institutes for Food and Drug Control, Beijing 102629, China
[2] State Key Laboratory of Component-Based Chinese Medicine, Tianjin University of Traditional Chinese Medicine, Tianjin 301617, China
* Correspondence: weilei8910@tjutcm.edu.cn (W.L.); masc@nifdc.org.cn (S.M.)

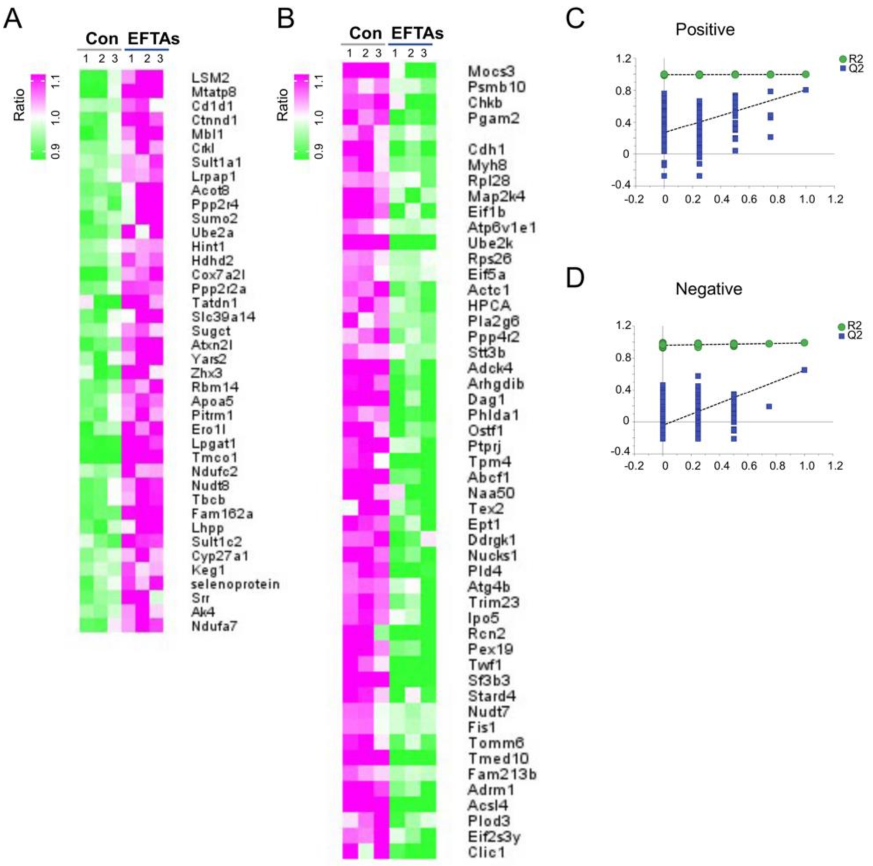

Figure S1. (A) The heatmap of upregulated proteins in hepatic proteomics. (B) The heatmap of downregulated proteins in hepatic proteomics. (C) The permutation test of OPLS-DA model in positive mode. (D) The permutation test of OPLS-DA model in negative mode.

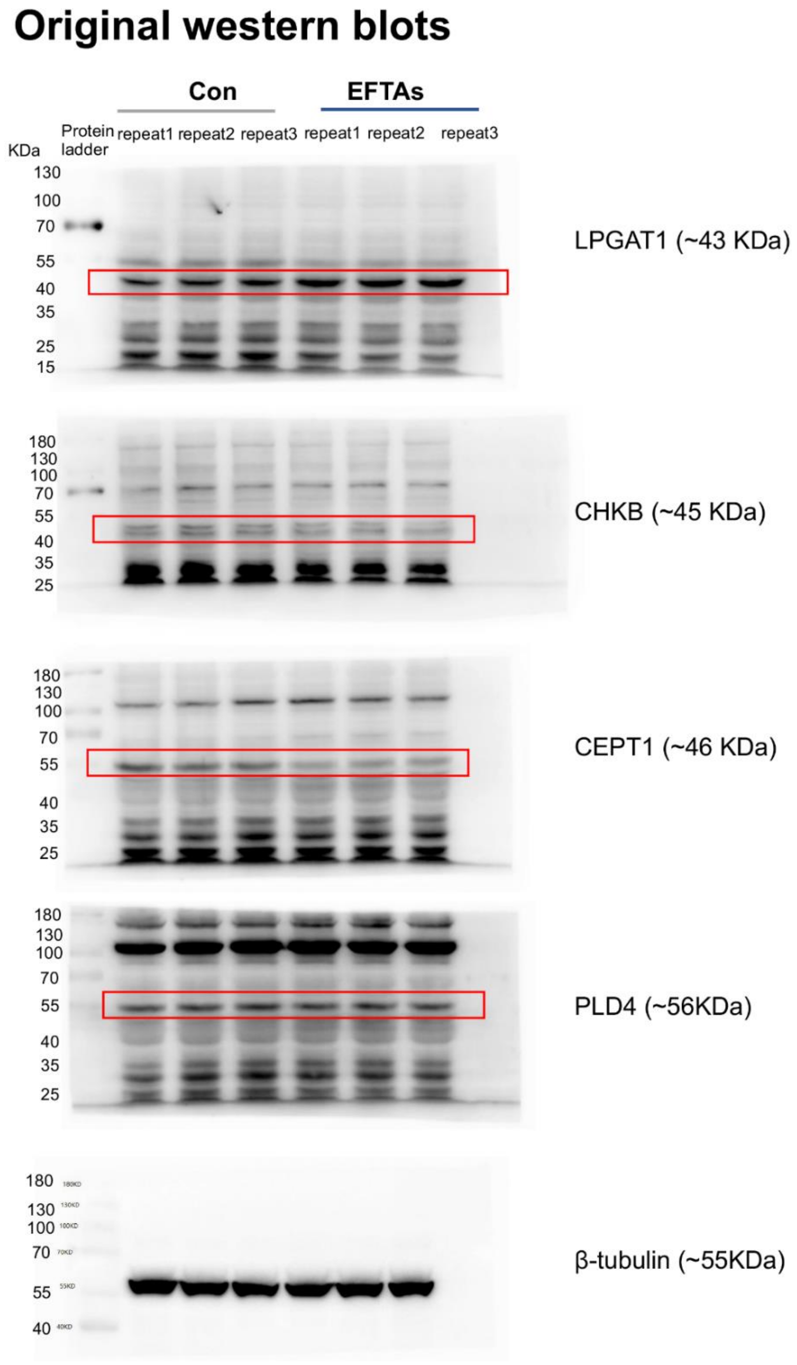

Figure S2. The original western blots of LPGAT1, CHKB, CEPT1 and PLD4.

Table S1. The identification of pyrrolizidine alkaloids in *E. fortunei*.

No.	ingredients	t_R/min	Composition	MS$^{(+)}$	MS/MS$^{(+)}$
1	Intermedine	18.62	$C_{15}H_{25}NO_5$	300.1	94.0, 138.1
2	Lycopsamine	19.63	$C_{15}H_{25}NO_5$	300.0	94.1, 138.1
3	Rinderine	20.22	$C_{15}H_{25}NO_5$	300.1	138.2, 156.2
4	Echinatine	20.61	$C_{15}H_{25}NO_5$	300.2	138.2, 156.2
5	Rinderine *N*-oxide	22.25	$C_{15}H_{25}NO_6$	316.2	138.0, 226.2
6	Echinatine *N*-oxide	22.78	$C_{15}H_{25}NO_6$	316.1	172.0, 138.1
7	Intermedine *N*-oxide	23.19	$C_{15}H_{25}NO_6$	316.2	172.1, 94.0
8	Lycopsamine *N*-oxide	23.85	$C_{15}H_{25}NO_6$	316.2	172.0, 138.1

Table S2. The differential metabolites identified in hepatic metabolomics.

No	Compounds	RT (min)	Mass (g/mol)	Precursor (g/mol)	Formula	VIP	*p*-value	FC
1	1-[(4-Bromophenyl)sulfonyl]-L-proline	1.5411	334.965	333.9571	$C_{11}H_{12}BrNO_4S$	1.79	0.011	1.53
2	7-ketodeoxycholic acid	6.8162	406.2719	405.2648	$C_{24}H_{38}O_5$	1.57	0.032	0.40
3	Hyodeoxycholic acid	8.1198	392.2927	391.2854	$C_{24}H_{40}O_4$	1.56	0.033	0.55
4	Formimidoyl-fortimicin A	10.2227	432.2696	387.2751	$C_{18}H_{36}N_6O_6$	1.68	0.029	0.56
5	gamma-Linolenic acid	10.472	278.2246	277.2185	$C_{18}H_{30}O_2$	1.68	0.020	0.64
6	N-{4-[Acetyl(methyl)amino]phenyl}-2-chloroacetamide	6.6738	240.0666	275.0361	$C_{11}H_{13}ClN_2O_2$	1.75	0.011	1.93
7	Palmitoleic acid	10.796	254.2246	507.4413	$C_{16}H_{30}O_2$	2.02	0.005	0.61
8	MG(0:0/18:2(9Z,12Z)/0:0)	10.505	354.277	389.2463	$C_{21}H_{38}O_4$	1.63	0.014	0.492
9	Taurohyodeoxycholic acid	6.3928	499.2968	498.2892	$C_{26}H_{45}NO_6S$	1.63	0.025	0.306
10	2-(1-Piperidinyl)-1H-benzimidazole	3.8667	201.1266	236.092	$C_{12}H_{15}N_3$	1.65	0.022	1.53
11	3,7-Dihydroxy-12-oxocholanoic acid	6.8162	406.2719	441.241	$C_{24}H_{38}O_5$	1.56	0.034	0.41
12	9,10-Epoxystearic acid	9.2091	298.2508	297.2436	$C_{18}H_{34}O_3$	2.01	0.0039	0.59
13	5,6-DHET	10.472	338.2457	359.2202	$C_{20}H_{34}O_4$	1.70	0.018	0.66
14	7-Ethyl-10-(4-N-aminopentanoic acid)-1-piperidino)carbonyloxycamptothecin	5.3499	618.269	599.2596	$C_{33}H_{38}N_4O_8$	1.46	0.047	0.47
15	4-Hydroxyphenylacetic acid	3.1023	152.0473	107.05	$C_8H_8O_3$	1.99	0.0045	1.838
16	2-Hydroxy Estrone	5.9771	513.276	512.2683	$C_{26}H_{43}NO_7S$	1.58	0.029	0.27
17	Tauroursodeoxycholic acid	5.9076	499.2968	498.288	$C_{26}H_{45}NO_6S$	1.66	0.015	0.29
18	Glycolaldehyde	1.1375	60.0211	112.0227	$C_2H_4O_2$	2.28	0.0002	2.32
19	Ursodeoxycholic Acid	7.0738	392.2927	451.3064	$C_{24}H_{40}O_4$	1.59	0.027	0.42
20	Quercetin	5.5035	302.0427	321.0432	$C_{15}H_{10}O_7$	1.80	0.007	0.55
21	N-Glycolylneuraminic acid	1.2993	325.1009	360.0706	$C_{11}H_{19}NO_{10}$	1.54	0.029	2.22
22	beta-Doradecin	8.694	580.3916	579.3901	$C_{40}H_{52}O_3$	1.76	0.020	2.60
23	Tuftsin	7.7219	500.3071	499.3054	$C_{21}H_{40}N_8O_6$	1.6432	0.027020529	2.69
24	Thr-Lys-Pro-Arg	7.1071	500.3071	499.3053	$C_{21}H_{40}N_8O_6$	1.55	0.035	1.89
25	Ser-His-Val-Lys	6.8162	469.2649	468.2602	$C_{20}H_{35}N_7O_6$	1.53	0.037	0.41
26	Phoenicoxanthin	9.4168	580.3916	579.3902	$C_{40}H_{52}O_3$	1.58	0.038	1.81
27	Oxidized Photinus luciferin	11.3111	249.9871	248.973	$C_{10}H_6N_2O_2S_2$	1.95	0.004	1.56
28	Oleoylglycerone phosphate	7.9462	434.2433	450.2636	$C_{21}H_{39}O_7P$	1.79	0.015	0.62
29	N-Benzoyl-N'-(4-methoxyphenyl)thiourea	5.5035	286.0776	241.0866	$C_{15}H_{14}N_2O_2S$	1.68	0.0135	0.51
30	N-(2-Cyanophenyl)-4-fluorobenzamide	10.5717	240.0699	239.0632	$C_{14}H_9FN_2O$	1.63	0.030	0.63
31	Lys-Lys-OH	11.6533	382.1852	381.174	$C_{12}H_{26}N_4O_3$	1.53	0.038	0.35
32	LPE(16:0/0:0)	8.6691	453.2855	905.5649	$C_{21}H_{44}NO_7P$	1.75	0.016	0.61
33	Kaempferol 3-[6''-(3-hydroxy-3-methylglutaryl)glucoside]-7-glucoside	0.7925	754.1956	753.1879	$C_{33}H_{38}O_{20}$	2.06	0.004	0.549
34	Ile-Pro-Ala-Asp-Val	5.9729	513.2799	594.2692	$C_{23}H_{39}N_5O_8$	1.51	0.038	0.44
35	Gly-Leu-Arg	11.6518	344.2172	379.1788	$C_{14}H_{28}N_6O_4$	1.71	0.017	0.32
36	Gly-Asp-Ile-Val-Ile	6.11	515.2955	596.2852	$C_{23}H_{41}N_5O_8$	1.65	0.022	0.41
37	Glutathione Reducedform	0.9753	307.0838	306.0769	$C_{10}H_{17}N_3O_6S$	1.72	0.013	0.58
38	Ferroxamine	6.4007	613.2649	612.2524	$C_{25}H_{45}FeN_6O_8$	1.54	0.029	0.55
39	Dimethyl 2-galloylgalactarate	0.859	390.0798	471.0604	$C_{15}H_{18}O_{12}$	1.46	0.042	0.51
40	Carmamycin A	5.5533	515.3029	514.2852	$C_{25}H_{45}N_3O_6S$	1.55	0.036	0.44
41	CID 101493123	12.1137	374.2668	596.3907	$C_{20}H_{38}O_6$	1.96	0.0032	1.62
42	1-Oleoylglycerophosphoinositol	12.1087	598.3118	619.2877	$C_{27}H_{51}O_{12}P$	1.64	0.028	1.66
43	(3R,4S,9R,11R)-29-(4-hydroxyphenyl)-4-methylnonacosane-3,9,11-triol	10.472	562.4961	583.4701	$C_{36}H_{66}O_4$	1.86	0.009	0.57

RT, retention time; VIP, variable influence in projection; FC, fold change.
